# Sexual self-concept, functioning, and practices of women with binge eating episodes

**DOI:** 10.1007/s40519-023-01565-0

**Published:** 2023-04-17

**Authors:** Geneviève Manuela Martin, Jérôme Tremblay, Marie-Pierre Gagnon-Girouard

**Affiliations:** 1 Department of Psychiatry and Neurosciences, Faculty of Medicine, Université Laval, 1050 Avenue de la Médecine, G1V 0A6 Québec, Canada; 2 Department of Sexology, Université du Québec à Montréal, Montréal, Canada; 3 Department of Psychology, Université du Québec à Trois-Rivières, Trois-Rivières, Canada

**Keywords:** Sexuality, Sexual self-concept, Sexual function, Sexual practices, Binge eating, Eating disorders

## Abstract

**Purpose:**

Positive sexuality has received little empirical attention in relation to eating disorders. Two tendencies related to sexuality have been identified among women with anorexia nervosa (avoidance) and bulimia nervosa (disinhibition), but it is unclear if they also apply to women with binge eating episodes without compensatory behaviors. This study aimed at (1) exploring the sexual self-concept, functioning, and practices of women with binge eating episodes with or without comorbid restrictive and/or compensatory behaviors, considering past experiences of violence, and (2) verifying the presence of distinct profiles of sexual dispositions among this population.

**Methods:**

In total, 253 women reporting recurrent episodes of loss of control related to food intake in the past 5 years, completed a web-based questionnaire. Descriptive and correlational analyses were conducted to outline participants’ sexual self-concept, functioning, and practices and to examine the relationship between these factors. A two-step cluster analysis was also performed to determine whether participants presented distinct profiles of sexual dispositions.

**Results:**

Participants were generally characterized by a negative sexual self-concept and poor sexual functioning. While a first subgroup of participants displayed a pattern of sexual difficulties and avoidance, a second subgroup had a positive sexual self-concept, better sexual functioning and a wider range of sexual practices. Subgroups did not differ relative to binge eating.

**Conclusions:**

Sexuality offers a platform for positive embodiment, which can lead to the improvement of body image and mind–body connection and may thus constitute an essential clinical target to improve treatment related to binge eating episodes.

*Level of evidence:* Level II: The experimental study is a non-randomized controlled trial.

## Background

Despite playing a key role in physical and psychological health and well-being, sexuality has received little empirical attention in relation to eating disorders (EDs) [1, 2]. While the limited research conducted to date signals the presence of sexual difficulties among individuals with an eating disorder (ED) [3–5, 11], it has examined sexuality through the prism of pathology [5, 11, 14, 15, 29] and offers little ground for the promotion of sexual health and well-being. In addition, it is unclear if these difficulties apply to all individuals with binge eating episodes, as studies almost exclusively concern anorexia nervosa (AN) and bulimia nervosa (BN). Yet binge eating is present across all ED diagnostic categories, and various sexual dispositions and experiences have been associated with problematic eating behaviors rather than diagnoses [11]. Quality of life and well-being-related factors that are specific to binge eating without compensatory behaviors and that constitute protective factors or levers of change in treatment remain poorly understood. Sexuality could provide a platform for the rehabilitation of individuals with an ED, as clinical features of these disorders intersect with factors that are associated with the onset and maintenance of sexual difficulties [5–9].

### What is known about sexuality and eating disorders

Compared to the general population, women with an ED are distinguished by poorer sexual function, and greater sexual anxiety, dissatisfaction and avoidance [10–14, 65]. Factors that are inherent to EDs, such as hormonal disruptions, weight loss and gain, and body image disturbances, are also known to influence sexual function and well-being [6, 12, 15–19]. Among women with persistent EDs, sexual adaptation has also been shown to be compromised [6, 15].

Two tendencies related to sexuality emerge from studies involving women with an ED: disinvestment and disinhibition. The disinvestment of sexuality is manifested in fewer sexual fantasies and lower sexual desire and satisfaction [5, 12, 14], and tends to be associated with restrictive eating behaviors [11]. Among women with AN, those with restrictive symptoms have been found to report a later age of onset (between 1 and 4 years) of masturbation and first consensual sexual intercourse than those reporting binge-compensatory behaviors [14, 20–22]. Available data suggest that women with AN are sexually active but many rate sexuality as uninteresting, unpleasant, or even disgusting [23, 24]. As yet, the motivation to engage in sexual contacts has not been studied. To date, no clear relationship has been identified between body mass index (BMI) and sexual function [5]. However, poorer sexual functioning distinguishes women with AN—in particular those with food restriction behaviors—from women with BN [11, 75] and women from the general population [11]. Moreover, a high frequency of sexual difficulties, which are often reported by women with AN, has been shown to predict poorer sexual functioning (e.g., sexual anxiety) and treatment outcome (e.g., chronicity of ED, treatment duration) [4, 12].

Disinhibition is expressed through sexual precociousness, promiscuity and risk-taking [4, 7, 25, 26]. It is more commonly documented in BN [27, 28]. By comparison with women with AN, women with BN report a variety of sexual fantasies that is similar to normative samples [14], higher sexual self-esteem and greater sexual desire [12, 17]. In a study by Weiderman et al. [22], the presence of a diagnosis of BN significantly predicted women’s involvement in both masturbation and sexual intercourse after controlling for age and BMI. This was not the case for AN. Nonetheless, BN has been found to predict the experience of sexual difficulties (arousal, lubrication, pain) and to be characterized by marked sexual dissatisfaction compared to women from the general population [9, 11]. Body image disturbances appear to influence the sexuality of women with BN. Indeed, body dissatisfaction is associated with lower sexual desire [75], and self-consciousness during sexual intercourse emerges as a potent predictor of BN symptoms [76]. Among women from a non-clinical sample, negative body esteem is associated with a greater tendency to dissociate during partnered sexual contacts [78]. Women reporting a greater tendency toward binge eating and dissociation during sexual activities with a partner displayed higher levels of cortisol in response to sexual stimuli. Impulsivity, which has been identified as a correlate of compensatory behaviors, increases sexual behaviors among women with binge eating [27]. It has been identified as a potential mediator of the relationship between a high prevalence of sexual risk-taking behaviors and disordered eating, especially in the case of BN [8]. This relationship needs to be empirically verified.

It is not possible, based on the available data, to determine if either or both tendencies related to sexuality observed in individuals with AN and BN, apply to binge eating in general. A greater frequency of binge eating episodes has been found to be associated with poorer sexual function, fewer orgasms, and more sexual dissatisfaction [29]. Furthermore, binge eating increases the risk of exhibiting a higher weight by three to six times. As the copresence of a higher weight and EDs has been linked to low sexual functioning, it is likely that at least a proportion of women with binge eating are characterized by negative sexual self-perceptions and/or experiences [16, 30, 31]. This claim is supported by findings from a study conducted with sexually active women, showing that women with BED + obesity displayed lower sexual function compared to those with no BED + obesity, and controls [29]. Interestingly, women with BED + obesity who reported multiple partners were more sexually dissatisfied. Shape concern and impulsivity were identified as predictors of low sexual function only among women with BED + obesity. As for women displaying a higher weight (in the presence or absence of BED), sexual function was predicted by emotional eating. As evidenced by these findings, further studies are needed to circumscribe the role of impulsivity, body image, and emotion regulation—all core features of EDs—in the sexuality of women with binge eating.

It is surprising that sexuality has not been explored as a potential lever in treatment change [11, 16, 32], considering that most women with an ED report being uncomfortable, preoccupied or dissatisfied with their body [33–36]. Body image disturbances have well-known negative consequences on sexual desire, arousal, pleasure and satisfaction in the general population [37–41] and may indeed influence the sexual self-concept, the choice of partners, as well as relational and sexual dynamics. These tendencies may be exacerbated by a history of sexual victimization or trauma [21, 67], which are risk factors for the development of an ED [16, 42], and have been shown to negatively affect the sexual self-concept and function of women with an ED [11, 29, 45] and women from the general population [43, 44, 74]. Among both groups, experiences of sexual victimization have notably been linked to an overinvestment of sexuality (referred to as hypersexuality, compulsive sexual behaviors, or lack of control in sexuality) and sexual risk-taking behaviors [70–73, 77]. The overinvestment of sexuality among women with binge-compensatory symptoms has been tied to emotion dysregulation, emotional eating, and psychiatric symptoms [66]. This suggests that sexuality may be used, namely, to regulate, neutralize or compensate for negative emotions, and/or in an attempt to feel better or regain control of oneself or the situation. Compared to the general population, individuals with an ED indeed report higher rates of all forms of childhood maltreatment [46, 47]. Moreover, the experience of multiple forms of childhood maltreatment has been shown to influence the development of symptoms of EDs [42]. The unveiling of a history of sexual victimization or trauma still remains the chief reason for broaching sexuality in evidence-based treatments [5, 16].

Going beyond pathological aspects of sexuality to fill the knowledge gap regarding the sexuality of women with binge eating episodes, the main objective of the present study is to explore the sexual self-concept, functioning, and practices (also referred to as “sexual dispositions”) of women with binge eating episodes. The secondary objective is to verify the presence of distinct profiles of sexual dispositions and compare these profiles in terms of sexual experiences and practices, binge eating severity and BMI.

## Methods

### Participants

Participants were 253 females self-reporting recurrent and sustained episodes of loss of control related to food intake, marked by the consumption of a large quantity of food and the presence of a feeling of being unable to stop or to refrain from eating, in 5 years prior to the study. Participants included in the study indicated being 18 years or older and having repeated episodes of binge eating (episodes associated with BN were not discriminated). Symptoms were self-reported, and no formal diagnosis of binge eating disorder was made. While both male and female participants were recruited, the number of males was insufficient (*n* = 6) to merit inclusion in the study. Prior engagement in sexual intercourse or a romantic relationship (defined as an intimate relationship with a partner for a minimum of 6 months) was not necessary for inclusion in the study. The exclusion criteria were: (1) age below 18 years and (2) the absence or non-recurrence of binge eating episodes during the previous 5 years. No participant needed to be removed from the sample based on the presence of exclusion criteria. In addition, individuals who reported being currently underweight (BMI under 18.5 kg/m^2^) were removed (*n* = 1) to exclude individuals who might present with AN.

### Procedure

Participants were recruited via email and Facebook/Instagram ads through the clientele of a community and a private clinic that offer help to individuals with ED. The recruitment ad specified that the study targeted individuals with behaviors fitting binge eating disorder and included a description of binge eating. Participants completed a questionnaire on an online platform (Qualtrics Research Suite), available between October 2020 and July 2021. All participants gave informed consent by clicking on a button indicating that they had read the study description, agreed to participate voluntarily in the study, and were 18 years or older. They were then redirected to the questionnaires that required approximately 50 min to complete. At the start of the questionnaire, participants were asked whether they had experienced frequent and sustained episodes of binge eating in the last 5 years. If they indicated that they had not, they were automatically redirected at the end of the questionnaire and thanked for their participation. This provided an additional safeguard against the inclusion of individuals who did not meet the binge eating criterion. The questionnaires were started 405 times and completed by 62.47% participants. Ethical approval for the project was obtained from the Ethics Committee for Research in Health Sciences of the Université Laval (September 8, 2020, #2020-230/08-09-2020).

### Measures

#### Sociodemographic and sexorelational profile

A brief self-report questionnaire was designed to establish participants’ profile (e.g., number of years of education, age at first sexual interests). As part of the questionnaire, participants had to specify if they presented one or more of the following conditions: (a) anxiety disorder; (b) depressive disorder; and (c) attention deficit and hyperactivity disorder. In addition, they were asked to indicate whether they had experienced neglect, direct (bullying; physical, psychological, and sexual violence) and indirect violence, before the age of 16.

#### Body mass index

Participants provided their height and weight, which were used to calculate BMI.

#### Binge eating

Participants had to signal the occurrence of frequent and continuous episodes of the following eating problems, in the previous 5 years: (a) loss of control of food intake followed by a feeling of guilt (behaviors akin to binge eating); (b) loss of control of food intake followed by compensatory behaviors akin to bulimia (e.g., use of laxatives and/or diuretics, excessive exercise, induced vomiting); and (c) food restriction (behaviors akin to anorexia).

In addition, the Binge Eating Scale [48] was used to assess the severity of binge eating behaviors. Items rated on a four-point response scale (0–4). Summed scores between 18 and 26 represent moderate binge eating and scores of 27 or more indicate severe binge eating. The scale showed a good internal consistency (Cronbach’s α = 0.88) within the present sample.

#### Sexual self-concept

The Multidimensional Sexual Self-Concept Questionnaire [50] was used to assesses 11 dimensions of the sexual self-concept: sexual consciousness; self-efficacy; assertiveness; optimism; satisfaction; monitoring; preoccupation; anxiety; depression; internal sexual control; and fear of sexuality. Items from each scale are rated on a five-point Likert-type scale (1 through 5), with higher averaged scores reflecting either well-constructed or deficient dimensions of the sexual self-concept, depending on the positive or negative nature of the scale. Reliability coefficients of the 11 scales are good to excellent, with alpha scores ranging between 0.85 and 0.92 for the present study, except for internal sexual control (α = 0.65). The 10 items of the Sexuality Scale assessing sexual self-esteem were also used [51] and exhibited excellent internal consistency (Cronbach’s α = 0.93) in the current sample.

#### Sexual functioning

To control for the presence of prevalent physical and mental conditions in EDs that are known to influence sexuality, participants had to indicate whether they presented one or many of the following: (a) thyroid gland problems; (b) diabetes; (c) polycystic ovary syndrome; (d) depressive symptoms; (e) anxiety symptoms; (f) attention deficit disorder with/without hyperactivity.

Sexual function was assessed using the Arizona Sexual Experience Scale [52]. The intensity of sexual desire, the capacity for arousal, lubrication and orgasm, and the level of satisfaction with orgasms, are rated on a six-point Likert-type scale (0 through 5). Higher scores reflect more sexual difficulties. In the current sample, the items yielded adequate internal consistency (Cronbach’s α = 0.74). Two scales of the Derogatis Sexual Functioning Inventory [53] were used to evaluate the variety of sexual fantasies and sexual satisfaction. They had good (Cronbach’s α = 0.81) to acceptable (Cronbach’s α = 0.65) internal consistency with the study sample. The Sexual Pleasure Scale [54] measures the level of sexual pleasure derived from sexual intimacy, contacts, and intercourse in romantic relationships, on a six-point Likert-type scale (0 through 5). High scores reflect greater sexual pleasure. The scale presents good internal consistency (Cronbach’s α = 0.88) with the study sample. The Sexual Motivation Scale [55] was used to measure intrinsic, integrated, identified, introjected, and external regulation, as well as amotivation, on a 7-point Likert-type scale (1 through 7). Higher mean scores reflect greater regulation or amotivation. Reliability coefficients on the six subscales are good to excellent (Cronbach’s α = 0.78–0.94) for the study sample. Two scales, rated on a five-point Likert-type scale (1 through 5), were designed to assess sexual avoidance and relationship to the body in sexuality (higher scores = negative relationship). The scales exhibited good internal consistency (Cronbach’s α = 0.82 and 0.81) with the study sample.

#### Sexual practices

The Sexual Practices Scale [56] was adapted for adults in the present study. It assesses the frequency of 20 autoerotic and partnered sexual practices in the previous 12 months, on a six-point Likert-type scale (0 trough 5). The Sexual Risk Survey [57] was used to assess the frequency of engagement in sexual behaviors that increase the risk of involuntary pregnancy or STIs in the past 6 months. The reliability coefficient of the scale with the present sample is good (Cronbach’s α = 0.85).

### Statistical analyses

Descriptive analyses were conducted to outline participants’ sexual self-concept, functioning, and practices and correlational analyses to examine the relationship between dimensions of the sexual self-concept and aspects of sexual functioning. *t* tests and ANOVAs were performed to determine if differences existed between subgroups of participants. To verify the presence of distinct profiles in the sample, a two-step cluster analysis was performed. Given the exploratory nature of the study, this analysis allowed the detection of homogenous groupings based on similar dispositions toward sexuality. Such groupings could then be compared to the tendencies reported in the literature related to sexual disinvestment and sexual disinhibition. The analysis included the following dimensions of the sexual self-concept, which had previously been standardized into z-scores: sexual self-esteem, efficacy, assertiveness, satisfaction, and anxiety. First, cases were pre-clustered using a sequential approach based on the definition of dense regions in the analyzed attribute-space. Second, the pre-clusters were merged statistically in a stepwise way until all clusters formed one cluster [58]. The optimal number of clusters was determined using the Bayesian information criterion (BIC). While models with the lowest BIC are preferred, models with two, three, and four clusters with the lowest BIC were contrasted [59]. Profiles were then compared on the remaining dimensions of the sexual self-concept, aspects of sexual functioning, sexual experience, BMI, and binge eating severity, using *t* tests. Considering the high number of comparisons that were tested, the Bonferroni correction suggests that only *p* values < 0.0015 should be considered as statistically significant.

## Results

Participants were aged between 18 and 65 years (*M* = 33.61, SD = 10.02) and reported an average BMI of 32.58 (SD = 9.46). More precisely, over three quarters of participants reported a BMI that could be categorized as overweight (21.2%) or obese (62.2%). In the previous 5 years, 47.04% had experienced frequent and continuous episodes of binge eating, in the absence of comorbid ED symptoms. Binge eating was reported with comorbid food restriction by 24.51% participants, binge-compensatory behaviors by 6.32%, and restrictive and binge-compensatory behaviors by 22.13%. The average BMI was significantly higher for participants reporting binge eating episodes solely (*M* = 35.58, SD = 9.38), compared to those with additional food restriction (*M* = 30.50, SD = 8.36), and food restriction and binge-compensatory behaviors (*M* = 27.98, SD = 8.47), *F*(3, 244) = 10.63, *p* < 0.001, η^2^ = 0.12, 95% CI [0.04, − 0.19]). No difference was found relative to BMI with participants presenting comorbid binge-compensatory behaviors. Binge eating severity was moderate in the sample (*M* = 25.0, SD = 9.23), based on the Binge Eating Scale criteria [48, 49]. Participants self-reported the following comorbid conditions: 48.61% anxiety; 28.46% depression; 25.45% ADHD; 13.45% thyroid gland problems; 11.45% polycystic ovary syndrome; and 3.69% diabetes.

Most participants were Canadian citizens (94.5%) born in North America (87.4%), identifying as cisgender females. They had completed a college (38.50%) or undergraduate degree (51.19%) and were working part- (13.44%) or full- (54.55%) time. Among those holding a job, the median annual salary before taxes was between $40,000 and $49,000 CAD. The majority reported being sexually attracted exclusively (41.90%) or mainly (39.92%) to males (5.93% = to males and females equally, 1.98% = mainly or exclusively to females, 8.30% = to persons regardless of their gender, 1.58% = were questioning their sexual orientation, and 0.40% = had no sexual attraction). At the time of the study, 61.27% were in a romantic relationship, 34.79% were single, separated, divorced, or widowed, and 3.95% reported other relational statuses.

Participants reported that they first became interested in sexuality on average at 11.71 years (SD = 3.61). Exposure to pornographic sexual content occurred 2 years later (*M* = 13.73 years, SD = 3.89). At the time of the study, 94.86% of participants indicated they had experienced at least one consensual sexual intercourse involving penetration in their lifetime. The first consensual sexual relationship occurred at a mean age of 16.70 years (SD = 3.54, range = 11–40). The majority (93.68%) of participants reported having been involved at least once in a romantic relationship lasting minimum 6 months. The average lifetime number of romantic partners was 4.30 (SD = 6.77, *Mdn* = 3.0). Most had had uniquely (87.29%) or mainly (8.90%) male partners. Participants’ longest romantic relationship lasted on average 7.78 years (SD = 6.05). The majority (70.46%) were in a romantic relationship at the time of the study. They rated their level of satisfaction with their current romantic relationship at 6.6/10 (SD = 2.50). The rate did not vary significantly with the presence of symptoms of anxiety, depression, or ADHD.

Past experiences of violence were common. Whereas only 7.57% of participants reported no experience of direct or indirect violence before the age of 16, 20.95% reported one form of violence, 19.37% = two, 26.48% = three, 15.02% = four, and 10.28% = five. More precisely, 51.19% had witnessed violence perpetrated against a parental figure, 33.20% experienced neglect by a parental figure, 39.13% physical violence, 56.52% psychological violence, and 51.0% sexual violence by a person within or outside of the family. Moreover, 71.15% were bullied by a non-family member and 27.27% had bullied a non-family member. All victims of indirect violence also experienced direct violence.

### Sexual self-concept

Table 1 presents the means and standard deviations for each of the various dimensions of the sexual self-concept. Compared to available normative data (not based on statistically significant value), participants scored on average 0.5 standard deviation lower on positive dimensions (consciousness, self-esteem, self-efficacy, internal sexual control, assertiveness, optimism, and satisfaction) and higher on negative dimensions (monitoring, preoccupation, anxiety, fear, depression) of the sexual self-concept. The only significant difference found relative to comorbid ED symptoms concerned sexual preoccupation. Participants with binge-compensatory symptoms reported greater sexual preoccupation (*M* = 2.06, SD = 1.40) compared to those presenting strictly binge eating (*M* = 1.09, SD = 0.93) or with binge-restrictive (*M* = 1.12, SD = 0.97) symptoms, *F*(3, 249) = 5.62, *p* < 0.001, η^2^ = 0.06 [0.01–0.12]. Depressive symptoms were associated with lower internal sexual control (*M* = 2.38, SD = 0.83, *n* = 37 vs no depression: *M* = 2.66, SD = 0.74, *n* = 131, *t*(58.71) = 1.93, *p* = 0.023, *d* = 0.76, 95% CI [0.02–0.73]) and a greater fear of sexuality (*M* = 1.91, SD = 1.31, *n* = 40 vs no depression *M* = 1.46, SD = 1.18, *n* = 131, *t*(59.47) = − 1.96, *p* = 0.027, *d* = 1.21, 95% CI [− 0.73, − 0.02]).

**Table 1:** Descriptive statistics for dimensions of the sexual self-concept

Dimensions	*n*	*M*	SD	Normative data
Sexual consciousness	253	2.59	0.94	3.01
Sexual self-esteem	226	2.13	0.86	2.62
Sexual self-efficacy	252	2.50	1.03	2.63
Internal sexual control	252	2.59	0.73	2.94
Sexual assertiveness	252	1.94	1.06	2.16
Sexual optimism	252	2.53	0.95	2.90
Sexual satisfaction	252	1.99	1.15	2.57
Sexual monitoring	251	1.83	1.08	1.58
Sexual preoccupation	253	1.24	1.03	0.77
Sexual anxiety	252	1.91	1.17	1.21
Fear of sexuality	252	1.58	1.21	1.33
Sexual depression	252	1.88	1.22	0.94

A history of violence distinguished participants on some dimensions of the sexual self-concept. Women who witnessed indirect violence toward a parental figure reported greater sexual depression (*M* = 2.03, SD = 1.19, *n* = 129 vs *M* = 1.72, SD = 1.24, *n* = 122, *t*(246.64) = − 2.01, *p* = 0.023, *d* = 1.21, 95% CI [− 0.50, − 0.01]). Victims of bullying were characterized by more sexual depression (*M* = 1.98, SD = 1.23, *n* = 179 vs *M* = 1.63, SD = 1.16, *n* = 73, *t*(141.18) = -2.14, *p* = 0.017, *d* = 1.21, 95% CI [− 0.56, − 0.02]), as well as increased sexual monitoring (*M* = 1.93, SD = 1.09, *n* = 178 vs *M* = 1.59, SD = 1.02, *n* = 73, *t*(142.56) = − 2.30, *p* = 0.011, *d* = 1.07, 95% CI [− 0.59, − 0.04]) and preoccupation (*M* = 1.36, SD = 1.07, *n* = 180 vs *M* = 0.94, SD = 0.89, *n* = 73, *t*(251) = − 2.90, *p* = 0.002, *d* = 1.02, 95% CI [− 0.68, − 0.13]). Finally, those reporting experiences of psychological or sexual violence showed more fear of sexuality (psychological violence: *M* = 1.74, SD = 1.20, *n* = 143 vs *M* = 1.37, SD = 1.19, *t*(233.02) = − 2.45, *p* = 0.007, *d* = 1.20, 95% CI [− 0.56, − 0.06]); sexual violence: *M* = 1.77, SD = 1.31, *n* = 127 vs *M* = 1.40, SD = 1.06, *n* = 123, *t*(248) = − 2.42, *p* = 0.008, *d* = 1.20, 95% CI [− 0.56, − 0.06]).

### Sexual functioning

The means and standard deviations on aspects of sexual functioning are presented in Table 2. By comparison with available normative data [52–55], participants displayed lower sexual functioning, pleasure, and satisfaction, and greater external sexual motivation, and amotivation. They also obtained higher intrinsic, integrated, identified, and introjected sexual motivation scores. The majority of participants indicated having had the following sexual fantasy at least once: making love (97.8%); having oral–genital contacts (91.3%); attaining sexual stimulation through artificial means (83.0%); having homosexual fantasies or having more than one sexual partner at once (79.6%); wearing erotic clothing (76.5%); having sex in unusual positions (73.4%); having a sexual encounter with a forbidden partner (73.0%), and being tied up during sexual contacts (71.6%). Sexual functioning did not significantly vary according to the presence of a history of violence or symptoms of anxiety, depression, and ADHD.

**Table 2:** Descriptive statistics for aspects of sexual functioning

Aspects of sexual functioning	*n*	*M*	SD	Normative data
Sexual dysfunction	224	18.60	3.91	13.50
Sexual pleasure	219	10.84	3.53	20.66
Sexual fantasy	230	10.00	3.50	6.00
Sexual satisfaction	217	6.08	2.21	8.89
Sexual motivation				
Intrinsic	200	11.46	2.61	5.81
Integrated	200	9.72	3.42	4.20
Identified	200	10.25	2.98	4.92
Introjected	200	8.23	3.72	3.04
External regulation	200	7.14	3.59	2.09
Amotivation	200	3.50	3.49	1.38
Sexual avoidance	225	2.50	0.85	–
Relationship to the body in sexuality	225	3.32	0.87	–

Table 3 presents the correlations between dimensions of the sexual self-concept and main aspects of sexual functioning. Fear of sexuality, sexual anxiety, and sexual depression are associated positively with sexual dysfunction, and negatively with sexual pleasure and satisfaction. Opposite relationships were found for the positive dimensions of the sexual self-concept.

**Table 3:** Correlations for dimensions of the sexual self-concept and aspects of sexual functioning

		1	2	3	4	5	6	7	8	9	10	11	12	13	14	15
1	Sexual consciousness	–														
2	Sexual self-esteem	0.57**	–													
3	Sexual self-efficacy	0.75**	0.53**	–												
4	Sexual assertiveness	0.62**	0.62**	0.62**	–											
5	Sexual optimism	0.57**	0.49**	0.65**	0.51**	–										
6	Sexual satisfaction	0.65**	0.55**	0.75**	0.60**	0.67**	–									
7	Internal sexual control	0.45**	0.25**	0.48**	0.34**	0.35**	0.46**	–								
8	Sexual anxiety	− 0.52**	− 0.51**	− 0.62**	− 0.58**	− 0.71**	− 0.69**	− 0.22**	–							
9	Fear of sexuality	− 0.54**	− 0.62**	− 0.60**	− 0.55**	− 0.55**	− 0.65**	− 0.29*	0.68**	–						
10	Sexual depression	− 0.48**	− 0.48**	− 0.60**	− 0.55**	− 0.69**	− 0.78**	− 0.31**	0.83**	0.61**	–					
11	Sexual dysfunction	− 0.53**	− 0.46**	− 0.46**	− 0.47**	− 0.31**	− 0.44**	− 0.22**	0.39**	0.40**	0.35**	–				
12	Sexual pleasure	0.44**	0.36**	0.49**	0.39**	0.42**	− 0.52**	0.23**	− 0.40**	− 0.45**	− 0.43**	− 0.36**	–			
13	Sexual satisfaction	0.36**	0.43**	0.46**	0.52**	0.55**	0.61**	0.22**	− 0.58**	− 0.42**	− 0.61**	− 0.41**	0.43**	–		
14	Avoidance of sexuality	− 0.54**	− 0.55**	− 0.57**	− 0.59**	− 0.50**	− 0.52**	− 0.17*	0.64**	0.64**	0.55**	− 0.49**	− 0.51**	− 0.49**	–	
15	Relationship to the body in sexuality	− 0.42**	− 0.51**	− 0.49**	− 0.51**	− 0.47**	− 0.49**	− 0.18*	056**	0.53**	0.51**	0.33**	− 0.35**	− 0.44**	.61**	–

*p < 0.05. **p < 0.001

### Sexual experiences

Most participants reported being sexually active in the year prior to the study. Table 4 presents the frequency of autoerotic and partnered sexual practices. Participants varied greatly in their reported frequency of sexual fantasies: 4.89% = none, 30.22% = 1–2 times per year, 27.56% = 1–3 times per month, and 37.33% 1 or more times per week. While 74.67% of participants reported engaging in autoerotic masturbation minimally once a month, fewer (45.33%) watched pornographic material or used sex toys alone (46.67%). Sexting with non-partners was not common (15.56%).

**Table 4:** Frequency of autoerotic and partnered sexual practices

	*n*	Frequency (%)					
		Never	Once or twice	Once every 3 months	Once every 2 months	One to three times a month	Once a week or more
Having sexual fantasies	225	4.89	8.44	7.56	14.22	27.56	37.33
Masturbating: self	225	4.0	4.89	8.0	8.44	29.78	44.89
Masturbating: partner	224	14.73	14.73	6.25	12.50	31.25	20.54
Watching pornographic material: alone	225	20.89	14.22	8.89	10.67	26.22	19.11
Watching pornographic material: with partner	224	71.42	16.52	4.91	3.13	3.13	0.89
Sexting with partner	225	56.89	14.67	8.0	4.89	8.44	7.11
Sexting with a person other than the partner	225	58.22	13.33	6.67	6.22	8.0	7.56
Using sex toys: alone	224	25.33	10.22	11.11	6.67	21.78	24.89
Using sex toys: with partner	225	45.34	18.22	9.33	10.22	14.22	2.67
Having sexual intercourse with a partner	224	10.26	9.38	6.69	9.38	30.36	33.93
Filming or taking photographs of sexual partner during sex	225	89.80	8.44	0.44	0.44	0.44	0.44
Being filmed or photographed by partner during sex	225	86.23	9.78	1.78	1.33	0.44	0.44
Taking part in a live sexual video	224	84.38	10.27	4.02	0.89	0.45	0
Dominating the sexual partner	225	73.78	14.67	4.0	4.0	2.67	0.88
Being dominated by sexual partner	225	49.78	16.0	7.11	7.11	13.33	6.67
Partner swapping	225	96.0	1.78	0.89	0.44	0.89	0
Having sexual contacts with more than one partner at a time	225	88.44	8.89	1.33	0.89	0	1.78
Cheating on partner	225	87.56	8.44	1.33	0.89	0	1.78
Paying for sexual services	224	99.10	0.45	0.45	0	0	0

Participants who engaged in partnered sexual activities (90.67%) did so mostly (75.35%) with a single partner (range = 1–37). The most common sexual practices were sexual intercourse (89.74%) and masturbation of the partner (85.27%). Few had integrated accessories or technology-based sexual practices (viewing pornography, sexting, etc.) with partners. While 50.22% reported having been sexually dominated by their partner at least once, 73.78% had never dominated their sexual partner. A minority had cheated on their sexual partner (12.44%), participated in multipartnered sexual contacts (11.56%), swapped partners (4.0%), or paid for sexual services (0.9%) once or more. The majority (64.29%) indicated they had engaged in sexual intercourse minimally once per month in the previous year. Three quarters (75.56%) estimated the ideal frequency of partnered sexual intercourse at once a week but only 33.93% attained that threshold.

Autoerotic and partnered sexual practices were aggregated into total scores and the first was found to be associated with a history of sexual violence. Indeed, victims of sexual violence reported engaging in fewer autoerotic sexual practices (*M* = 2.91, SD = 1.15 vs no sexual victimization: *M* = 3.34, SD = 1.00, *t*(217.93) = 3.00, *p* = 0.002, *d* = 1.08, 95% CI [0.13, 0.67]). No other significant relationship was found for a history of violence. As for comorbid psychiatric symptoms, participants reporting symptoms of anxiety had significantly more partnered sexual practices (*M* = 2.79, SD = 1.36 vs no anxiety: *M* = 2.32, SD = 1.18, *t*(200.29) = − 2.63, *p* = 0.005, *d* = 1.27, 95% CI [− 0.64, − 0.09]).

Correlational analyses revealed small but significant associations between the number of forms of violence experienced and sexual monitoring (*r* = 0.14, *p* = 0.031), sexual preoccupation (*r* = 0.14, *p* = 0.029), fear of sexuality (*r* = 0.13, *p* = 0.036), sexual depression (*r* = 0.16, *p* = 0.012), age of first sexual interest (*r* = − 0.19, *p* = 0.003), and BMI (*r* = 0.15, *p* = 0.019) but not with binge eating severity.

### Profiles of sexual dispositions

Evaluation of the BIC revealed a 2-cluster (BIC = 582.62), a 3-cluster (BIC = 566.10) and a 4-cluster (BIC = 578.24) solution. The 3-cluster solution included the first initial cluster and the splitting of the second cluster into 2, while the 4-cluster solution consisted in the splitting in 2 of the two initial clusters. While the 3- and 4-cluster solutions had a lower BIC, the magnitude of the BIC change was not worth the reduction of parsimony and the difficulty of interpretation of clusters. The 2-cluster solution was retained based on its theoretical meaningfulness, parsimony, and explanatory power. The dimensions of the sexual self-concept used to define the profiles were: sexual self-esteem, efficacy, assertiveness, satisfaction, and anxiety. Sexual self-esteem and anxiety were included given their well-documented influence on sexual health and well-being [56]. The remaining dimensions were selected by examining the correlation matrix, which revealed a strong association between them and with sexual self-esteem and anxiety. Table 5 presents mean scores for the sexual self-concept dimensions and aspects of sexual functioning used to determine the clusters.

**Table 5:** Descriptive statistics of *t* test comparisons between profiles

	Cluster 1 Negative sexual dispositions (*n* = 113)	Cluster 2 Positive sexual dispositions (*n* = 113)	*t* or *X*^*2*^
Sexual self-concept			
Sexual consciousness	2.08 (.84)	3.17 (.62)	*t*(224) = 11.17, *p* < 0.001, *d* = 0.73
Sexual monitoring	1.91 (1.04)	1.76 (1.06)	*t*(222.91) = − 1.13, *p* = 0.13
Internal sexual control	2.39 (0.68)	2.86 (0.67)	*t*(223.98) = 5.32, *p* < 0.001, *d* = 0.67
Sexual optimism	1.94 (0.78)	3.13 (0.64)	*t*(215.72) = 12.57, *p* < 0.001, *d* = 0.71
Sexual depression	2.71 (0.91)	0.96 (0.78)	*t*(217.81) = − 15.68, *p* < 0.001, *d* = 0.84
Fear of sexuality	2.23 (1.12)	0.79 (0.70)	*t*(224) = − 11.66, *p* < 0.001, *d* = 0.93
Sexual preoccupation	1.11 (1.01)	1.40 (1.06)	*t*(223.40) = 2.09, *p* = 0.019, *d* = 1.04
Sexual functioning			
Sexual fantasies	9.31 (3.49)	10.93 (3.23)	*t*(213.39) = 3.55, *p* < 0.001, *d* = 3.36
Sexual difficulties	19.81 (3.69)	16.95 (3.37)	*t*(208) = − 5.88, *p* < 0.001, *d* = 3.54
Sexual pleasure	9.33 (3.27)	12.63 (2.70)	*t*(198.29) = 7.90, *p* < 0.001, *d* = 3.00
Sexual motivation: intrinsic	10.37 (3.29)	12.52 (0.80)	*t*(198) = 6.41, *p* < 0.001, *d* = 2.38
Sexual motivation: integrated	8.25 (3.82)	11.16 (2.15)	*t*(198) = 6.63, *p* < 0.001, *d* = 3.10
Sexual motivation: identified	9.47 (3.37)	11.00 (2.30)	*t*(198) = 3.77, *p* < 0.001, *d* = 2.88
Sexual motivation: introjected	8.00 (3.72)	8.46 (3.73)	*t*(197.94) = 0.87, *p* = 0.19
Sexual motivation: external regulation	8.22 (3.67)	6.07 (3.18)	*t*(192.97) = 4.43, *p* < 0.001, *d* = 3.43
Sexual motivation: amotivation	4.98 (3.81)	2.04 (2.38)	*t*(198) = − 6.56, *p* < 0.001, *d* = 3.17
Avoidance of sexuality	3.03 (0.78)	1.97 (0.54)	*t*(223) = 11.78, *p* < 0.001, *d* = 0.67
Relationship to the body in sexuality	3.76 (0.68)	2.88 (0.82)	*t*(223) = − 8.74, *p* < 0.001, *d* = 0.75
Sexual experience			
Age at first sexual interest	12.15 (3.76)	11.13 (3.46)	*t*(221.17) = − 2.12, *p* = 0.018, *d* = 3.61
Age at first consensual sexual relationship	17.05 (3.93)	16.20 (2.40)	*t*(224) = − 1.96, *p* = 0.025, *d* = 3.25
Number of sexual partners	1.43 (2.03)	2.30 (4.33)	*t*(224) = 1.93, *p* = 0.023, *d* = 3.38
Sexual practices (frequency over the last 12 months)			
Autoerotic	3.01 (1.14)	3.30 (0.98)	*t*(201.71) = 2.00, *p* = 0.023, *d* = 1.07
Partnered	2.23(1.32)	3.12 (1.36)	*t*(195.53) = 4.65, *p* < 0.001, *d* = 1.34
Ideal frequency of sexual intercourse	4.53 (0.89)	4.82 (0.63)	*t*(210) = 2.69, *p* = 0.004, *d* = 0.78
Engaging in risky sexual activities	6.70 (4.94)	9.26 (5.44)	*t*(189.49) = 3.43, *p* < 0.001, *d* = 5.19
History of violence (%)			
Indirect violence	56.63	48.21	*X*^2^(1, 226) = 1.60, *p* = 0.21, *φ* = 0.08
Neglect	33.63	36.38	*X*^2^(1, 226) = 0.18, *p* = 0.68, *φ* = − 0.03
Bullying	71.68	69.91	*X*^2^(1, 226) = 0.09, *p* = 0.77, *φ* = 0.02
Physical violence	38.94	40.71	*X*^2^(1, 226) = 0.74, *p* = 0.79, *φ* = − 0.02
Psychological	60.18	53.98	*X*^2^(1, 226) = 0.89, *p* = 0.35, *φ* = 0.06
Sexual	57.52	45.95	*X*^2^(1, 226) = 3.01, *p* = 0.08, *φ* = 0.12
Binge eating-related variables			
BMI	33.70 (9.99)	32.21 (9.17)	*t*(216.25) = − 1.16, *p* = 0.13, *d* = 9.58
Binge eating severity	25.82 (9.83)	24.01 (8.82)	*t*(160) = − 1.24, *p* = 0.22, *d* = 9.36

Based on the cluster analysis, the first group included 50.0% of the sample and was characterized by significantly lower mean scores on all positive dimensions of the sexual self-concept and higher sexual fear, depression, and monitoring. By contrast, the second group displayed significantly higher mean scores on all positive dimensions of the sexual self-concept and lower mean scores on all negative dimensions of the sexual self-concept, except for sexual preoccupation. The first group was labelled *Negative sexual dispositions,* while the second group that appears better disposed for the experience of sexuality was labelled *Positive sexual dispositions*.

Regarding sexual functioning, the first group reported significantly more sexual difficulties, less sexual pleasure and satisfaction, more amotivation and externally regulated sexual motivation, greater sexual avoidance and negative relationship to the body in sexuality. By contrast, the second group presented better sexual functioning and more internally regulated sexual motivation (except for introjected motivation).

Profiles were further distinguished based on sexual experience, sexual practices, and binge eating-related variables, using *t* tests. Participants in the positive sexual dispositions profile tended to report a lower age at first sexual interest and first consensual sexual relation but these differences were not significant. Participants were also inclined to report having had more sexual partners in the preceding 6 months and engaging in more autoerotic, partnered, and risky sexual practices.

Profiles did not vary significantly according to history of violence, BMI, binge eating severity, or the presence of restrictive or binge-compensatory behaviors, or comorbid physical or psychiatric conditions.

## Discussion

The study sheds light on a variety of positive aspects of the sexuality of women with binge eating episodes. We found that most participants were sexually active in the year prior to the study. The mean age of the first consensual sexual intercourse (*M* = 16.70, SD = 3.54) was comparable to the general population [56, 68] and to individuals displaying binge-compensatory behaviors [14, 22], but a larger variation was observed in the present sample. In addition, the range and frequency of sexual fantasies varied greatly among participants. Compared to normative samples, participants scored higher on all negative dimensions of the sexual self-concept. This was especially the case for women with a history of violence. Surprisingly, the great majority of the sample reported such experiences. Women who experienced two or more forms of violence were less positively disposed toward sexuality: they displayed greater sexual monitoring, preoccupation, depression, and fear of sexuality.

Although sexual functioning, pleasure and satisfaction were low, sexual motivation was high among participants. The findings concerning participants’ negative self-perceptions toward sexuality are coherent with the documented association between binge eating, low self-esteem and overwhelming bodily shame [60, 61]. Such perceptions may be rooted in past experiences of violence, which also contribute to the etiology of EDs. High motivation to engage in sexual activities for both personal and instrumental reasons suggests an underlying desire to experience sexual feelings and contacts. This supports the integration of sexuality as a prominent target in binge eating treatment.

In line with findings suggesting that women with binge eating differ in terms of relation to their body [62], we identified two distinct profiles among participants with binge eating episodes. The profiles did not vary with the presence of compensatory or restrictive behaviors. The first profile included women displaying a positive sexual self-concept (except for sexual preoccupation), better sexual functioning, and greater internally regulated sexual motivation. While women with BED + obesity have been found to exhibit lower sexual functioning compared to women with no BED + obesity and controls [29], this study points to a subgroup of women who have a more positive relationship with sexuality. These aspects of sexuality could be targeted in treatment to improve body image issues and provide positive embodiment experiences in this subgroup of women. Future studies should further investigate this profile and the demarcation between positive investment of sexuality and sexual disinhibition as observed in relation to BN [27, 28].

The second profile was comprised of women with a weaker sexual self-concept, more sexual difficulties, external sexual motivation, and a negative relationship to the body in sexuality. These sexual dispositions are akin to the sexual disinvestment tendencies of women with AN. The two profiles did not vary in terms of self-reported ED, BMI, depressive, anxious, and ADHD symptoms, and history of violence. This suggests that the severity of these factors may not account for the difference in sexual dispositions. Two mechanisms involved in binge eating may contribute to explaining this difference. First, cognitive restraint, i.e., the obsessive rumination about food even when it is not translated into actual restrictive behaviors, could contribute to distinguishing the profile more akin to AN. Indeed, women who are more obsessed with eating may be less invested in sexual fantasizing and less aware of their sexual needs and desires. Second, rumination about body image may interfere with the positive experience of sexuality and lead to sexual avoidance, which may in turn negatively affect the sexual self-concept. This must be verified in future studies integrating standardized measures of ED-specific and general psychiatric symptoms.

On the whole, the copresence of self-reported psychiatric symptoms did not have a marked impact on the sexual dispositions of women with binge eating episodes. Comorbid symptoms of depression were associated with lower sexual internal control, which is consistent with tendencies observed relative to general locus of control [63]. They were also linked to a higher fear of sexuality. Women with both binge eating episodes and mood-related symptoms may find sexual intimacy especially challenging, leading to the apprehension or fear of sexuality. Anxiety was associated with a wider variety of sexual practices with partners. For women with anxiety symptoms, sexuality may serve to release tension or to maintain their relationship through the sexual satisfaction of the partner. Given the high occurrence of psychiatric conditions in EDs and their known influence on sexuality, there is a need for further studies examining their interface with binge eating episodes and sexuality.

Some considerations limit the generalization of findings, notably the homogeneity of the sample (cisgender, heterosexual, white women, with a higher weight) and the absence of a formal ED diagnosis. Initial plans for recruitment and data collection targeted the clientele of a BED in-group treatment program at a community clinic specializing in the treatment of EDs. Due to the COVID-19 pandemic, these plans had to be adapted to an online platform. Whereas admission in BED treatment is based on a thorough 1-h interview that assesses whether participants meet all DSM-5 criteria for BED, this screening procedure could not easily be converted to an online self-report questionnaire. Nonetheless, close to half (47.04%) of the sample reported frequent binge eating episodes that were sustained over the previous 5 years, in the absence of comorbid ED symptoms. Most of them reported a higher weight, which is closely linked to binge eating, especially in the absence of compensatory behaviors, as it is the case in BED. In addition, the chronicity of binge eating episodes was targeted in inclusion criteria, but it was not defined in terms of episodes per week. Therefore, it is possible that individuals experiencing less frequent episodes of binge eating or repeated episodes of a limited duration, more akin to the diagnosis of Other Specified Feeding or Eating Disorder or to non-clinical binge eating, were incorporated in the sample. Sample composition is, therefore, not based on a clear diagnostic group. In addition, a significant percentage of the sample reported food restriction (24.51%), compensatory behaviors (6.32%), or both (22.13%) at least once in the last 5 years. The two profiles of sexual dispositions identified in the study did not vary significantly according to the presence of food restriction and/or compensatory behaviors, but it is nonetheless possible that the presence of symptoms overlapping other EDs influenced the findings. This issue constitutes an important limitation of this study in terms of internal validity. However, the present sample does reflect the natural clientele of community and private organizations that offer help to people with binge eating, which strengthens the external validity of the study. As initially planned, data collection included pharmacology as well as the use of standardized psychiatric measures for anxiety, depression, and ADHD, which are highly comorbid with EDs [79, 80]. To facilitate the completion of the online questionnaires in a timely manner, participants were instead asked to self-report the presence of those disorders, as well as thyroid problems, diabetes, and polycystic ovary syndrome. Future studies based on DSM-5 diagnostic groups must be conducted to delineate the sexual dispositions associated with binge eating, compare them with those related to AN and BN, and control the presence of a larger range of psychiatric symptoms. As information relative to pharmacotherapy could not be easily or accurately collected as part of the online adaptation of the project, it was altogether excluded. Given its known impact on sexuality, it should be controlled for in future studies on EDs. Considering the exploratory nature of the present study, comparisons were made using normative data, but future studies should integrate a control group.

Certain limitations associated with online studies must be considered in the interpretation of the present findings. First, the study was conducted as most of the population was confined or working from home, due to the sanitary measures imposed during the pandemic. While this situation may have increased the visibility of the publicity for the project, it is possible that the sample may not be entirely representative of non-internet users, individuals without high-speed internet, individuals who were highly solicited as part of their work outside of home (e.g., nurses), individuals living in rural areas, and individuals with less education or a lower income. Second, participants may have misrepresented their identity or characteristics, or answered in socially desirable ways. Third, individuals who respond to invitations to participate in online surveys—in this case without monetary compensation—may differ from those who do not. Fourth, the conditions in which participants completed the questionnaire could not be controlled, which may add statistical noise. Replication of the study, both in-person and online, could help reduce these potential biases.

Findings support the need to investigate how sexuality interacts with binge eating behaviors and body image issues in view of incorporating it in ED treatment. Since different profiles emerge from our results, more attention should be given to personal experiences of sexuality, while avoiding one-size-fits-all approaches to prevent any feelings of not being understood. For example, individuals with binge eating, especially those who are more positively disposed toward sexuality, sexual activities could be useful in providing embodied experiences of comfort and pleasure. Such experiences could fight against the shame and self-blame related to overeating behaviors that are fused within the person’s sense of identity [64]. In contrast, for individuals with a more difficult relation with sexuality, a trauma-informed approach may be more adequate in certain cases [81].

## Conclusion

This first study examining the sexual self-concept, functioning, and practices of women with binge eating episodes identified two subgroups worthy of further attention in future studies: one group presenting dispositions that are akin to those documented in women with AN, and another group more positively disposed toward sexuality. The sexual self-concept, and its interaction with a history of victimization, body image, and symptomatology, appear to be promising avenues for both research and treatment. Findings from the present study suggest that women with binge eating episodes are not all the same regarding sexuality, highlighting the importance of investigating how each relates to her own sexuality. Indeed, further investigation of the sphere of sexuality is needed to increase current understanding of binge eating, as sexuality could be used as a protective factor in the treatment of binge eating and negative body image.

### Strengths and limits

Findings from the study improve current understanding of the sexuality of women with binge eating episodes by expanding beyond pathology to include positive sexual dispositions. They show that on the whole, women with binge eating episodes show difficulties related to sexuality—some of which are associated with a history of violence—but a subgroup presents positive dispositions toward sexuality. The limits of the study are for the most part related to the adaptation of the in-person study to an online platform, due to the COVID-19 pandemic.

### What is already known on this subject?

Relationship difficulties are common among individuals with an ED, and they are tied to the manifestation of symptoms from those disorders. While most ED treatment programs target relationship functioning, few include sexuality. Yet, available studies suggest that sexuality is problematic among this clientele. To date, however, studies have focused almost exclusively on sexual dysfunctions among individuals with AN or BN. Therefore, the nature and extent of their sexual dispositions beyond pathology remains to be determined, and it is unclear whether the documented tendencies also apply to individuals with binge eating episodes without compensatory behaviors. A better understanding of the sexual dispositions of individuals with EDs is essential to identifying adaptive dispositions that can act as protective factors and increasing the efficacy of interventions.

### What this study adds?

As a result of this study, we know of the existence of a subgroup of women with binge eating episodes who present adaptive sexual dispositions akin to those found among women from the general population. This is an important contribution for research—which has omitted to consider positive sexuality—and treatment, as dispositions that promote sexual health and well-being can provide a base for tackling issues, such as body image, that are central to EDs.

## Data Availability

The data sets used and/or analysed during the current study are available from the corresponding author on reasonable request.

## References

[1] Mitchell KR, Lewis R, O’Sullivan LF, Fortenberry JD (2021). What is sexual wellbeing and why does it matter for public health?. Lancet Public Health.

[2] World Health Organization (2006). Defining sexual health—report of a technical consultation on sexual health 28–31 January 2002, Geneva.

[3] Cassioli E, Rossi E, Castellini G, Sensi C, Mancini M, Lelli L, Monteleone AM, Ricca V, Stanghellini G (2020). Sexuality, embodiment and attachment style in anorexia nervosa. Eat Weight Disord.

[4] Fichter MM, Quadflieg N, Hedlund S (2006). Twelve-year course and outcome predictors of anorexia nervosa. Int J Eat Disord.

[5] Price T, Zebitz M, Giraldi A, Lokind TS, Treasure J, Sjögren JM (2020). Sexual function and dysfunction among women with anorexia nervosa: a systematic scoping review. Int J Eat Disord.

[6] Castellini G, Franzago M, Bagnoli S, Lelli L, Balsamo M, Mancini M (2017). Fat mass and obesity-associated gene (FTO) is associated to eating disorders susceptibility and moderates the expression of psychopathological traits. PLoS ONE.

[7] Castellini G, Lelli L, Cassioli E, Ricca V (2019). Relationships between eating disorder psychopathology, sexual hormones and sexual behaviours. Mol Cell Endocrinol.

[8] Castellini G, Rossi E, Ricca V (2020). The relationship between eating disorder psychopathology and sexuality: etiological factors and implications for treatment. Curr Opin Psychiatry.

[9] Dunkley CR, Gorzalka BB, Brotto LA (2020). Associations between sexual function and disordered eating among undergraduate women: an emphasis on sexual pain and distress. J Sex Marital Ther.

[10] Arcelus J, Yates A, Whiteley R (2012). Romantic relationships, clinical and sub-clinical eating disorders: a review of the literature. Sex Relation The.

[11] Castellini G, Lelli L, Lo Sauro C, Fioravanti G, Vignozzi L, Maggi M (2012). Anorectic and bulimic patients suffer from relevant sexual dysfunctions. J Sex Med.

[12] Pinheiro AP, Raney TJ, Thornton LM, Fichter MM, Berrettini WH (2010). Sexual functioning in women with eating disorders. Int J Eat Disord.

[13] Raboch J, Faltus F (1991). Sexuality of women with anorexia nervosa. Acta Psychiatr Scand.

[14] Rothschild BS, Fagan P, Woodall C, Andersen AE (1991). Sexual functioning of female eating disordered patients. Int J Eat Disord.

[15] Castellini G, Lo Sauro C, Lelli L, Godini L, Vignozzi L, Rellini AH (2013). Childhood sexual abuse moderates the relationship between sexual functioning and eating disorder psychopathology in anorexia nervosa and bulimia nervosa: a 1-year follow-up study. J Sex Med.

[16] Castellini G, Lelli L, Cassioli E, Ricca V, Maggi M (2016). Sexuality in eating disorders patients: etiological factors, sexual dysfunction and identity issues. A systematic review. Horm Mol Biol Clin Investig..

[17] Morgan CD, Wiederman MW, Pryor TL (1995). Sexual functioning and attitudes of eating-disordered women: a follow-up study. J Sex Marital Ther.

[18] van Buren DJ, Williamson DA (1988). Marital relationships and conflict resolution skills of bulimics. Int J Eat Disord.

[19] Whisman MA, Dementyeva A, Baucom DH, Bulik CM (2012). Marital functioning and binge eating disorder in married women. Int J Eat Disord.

[20] Pryor T, Wiederman MW, McGilley B (1996). Clinical correlates of anorexia nervosa subtypes. The Int J Eat Disord.

[21] Schmidt U, Evans K, Tiller J, Treasure J (1995). Puberty, sexual milestones and abuse: how are they related in eating disorder patients?. Psychol Med.

[22] Wiederman MW, Pryor T, Morgan CD (1996). The sexual experience of women diagnosed with anorexia nervosa or bulimia nervosa. Int J Eat Disord.

[23] Carter FA, Carter JD, Luty SE, Jordan J, McIntosh VV, Bartram AF (2007). What is worse for your sex life: starving, being depressed, or a new baby?. Int J Eat Disord.

[24] Eckert ED, Halmi KA, Marchi P, Grove E, Crosby R (1995). Ten-year follow-up of anorexia nervosa: clinical course and outcome. Psychol Med.

[25] Irving LM (1990). Mirror images: effects of the standard of beauty on the self- and body-esteem of women exhibiting varying levels of bulimic symptoms. J Soc Clin Psychol.

[26] Kaltiala-Heino R, Rimpel M, Rissanen A, Rantaven P (2001). Early puberty and early sexual activity are associated with bulimic-type eating pathology in middle adolescence. J Adolesc Health.

[27] Culbert KM, Klump KL (2005). Impulsivity as an underlying factor in the relationship between disordered eating and sexual behavior. Int J Eat Disord.

[28] Quadflieg N, Fichter MM (2003). The course and outcome of bulimia nervosa. Eur Child Adolesc Psychiatry.

[29] Castellini G, Mannucci E, Mazzei C, Lo Sauro C, Faravelli C, Rotella CM (2010). Sexual function in obese women with and without binge eating disorder. J Sex Med.

[30] De Zwaan M (2001). Binge eating disorder and obesity. Int J Obes.

[31] Fairburn CG, Brownell KD (2002). Eating disorders and obesity.

[32] Tolosa-Sola I, Gunnard K, Giménez Muniesa C, Casals L, Grau A, Farré JM (2017). Body dissatisfaction and eating disorder symptomatology: which factors interfere with sexuality in women with eating disorders?. J Health Psychol.

[33] Ahrberg M, Trojca D, Nasrawi N, Vocks S (2011). Body image disturbance in binge eating disorder: a review. Eur Eat Disord Rev.

[34] Lewer M, Nasrawi N, Schroeder D, Vocks S (2016). Body image disturbance in binge eating disorder: a comparison of obese patients with and without binge eating disorder regarding the cognitive, behavioral and perceptual component of body image. Eat Weight Disord.

[35] Lewer M, Bauer A, Hartmann AS, Vocks S (2017). Different facets of body image disturbance in Binge Eating Disorder: a review. Nutrients.

[36] Velázquez López HJ, Vázquez Arévalo R, Mancilla Díaz JM (2018). Binge eating disorder in men. A review of the relevant variables in the literature. Salud Mental..

[37] Ackard DM, Kearney-Cooke A, Peterson C (2000). Effect of body image and self-image on women’s sexual behaviors. Int J Eat Disord.

[38] McCool-Myers M, Theurich M, Zuelke A, Knuettel H, Apfelbacher C (2018). Predictors of female sexual dysfunction: a systematic review and qualitative analysis through gender inequality paradigms. BMC Womens Health.

[39] Poggiogalle E, Di Lazzaro L, Pinto A, Migliaccio S, Lenzi A, Donini LM (2014). Health-related quality of life and quality of sexual life in obese subjects. Int J Endocrinol..

[40] Quinn-Nilas C, Benson L, Milhausen RR, Buchholz AC, Goncalves M (2016). The relationship between body image and domains of sexual functioning among heterosexual, emerging adult women. J Sex Med.

[41] Wallwiener S, Strohmaier J, Wallwiener LM, Schönfisch B, Zipfel S, Brucker SY (2016). Sexual function is correlated with body image and partnership quality in female university students. J Sex Med.

[42] Dion J, Boulianne-Simard C, Godbout N, Aimé A, Maïano C, Ricard MM (2020). Les troubles des conduites alimentaires.

[43] Guyon R, Fernet M, Canivet C, Tardif M, Godbout N (2020). Sexual self-concept among men and women child sexual abuse survivors: emergence of differentiated profiles. Child Abuse Negl.

[44] Wawrzyniec J, Spiegel S (2008). Sexual self-concept and sexually abused males: early data on the development of a scale. Int J Sex Health.

[45] Castellini G, Trisolini F, Ricca V (2014). Psychopathology of eating disorders. J Abnorm Psychol.

[46] Becker DF, Grilo CM (2011). Childhood maltreatment in women with binge-eating disorder: associations with psychiatric comorbidity, psychological functioning, and eating pathology. Eat Weight Disord.

[47] Molendijk ML, Hoek HW, Brewerton TD, Elzinga BM (2017). Childhood maltreatment and eating disorder pathology: a systematic review and dose-response meta-analysis. Psychol Med.

[48] Gormally J, Black S, Daston S, Rardin D (1982). The assessment of binge eating severity among obese persons. Addict Behav.

[49] Marcus MD, Wing RR, Hopkins J (1988). Obese binge eaters: affect, cognitions, and response to behavioral weight control. J Consult Clin Psychol.

[50] Snell WE Jr. In Snell WE Jr. New directions in the psychology of human sexuality: Research and theory. Cape Girardeau: Snell Publications; 2002. http://cstl-cla.semo.edu/snell/books/sexuality/sexuality.htm.

[51] Snell WE, Papini DR (1989). The Sexuality Scale: an instrument to measure sexual-esteem, sexual depression, and sexual preoccupation. J Sex Res.

[52] McGahuey CA, Gelenberg AJ, Laukes CA, Moreno FA, Delgado PL, McKnight KM (2000). The Arizona Sexual Experience Scale (ASEX): reliability and validity. J Sex Marital Ther.

[53] Derogatis LR, Melisaratos N (1979). The DSFI: a multidimensional measure of sexual functioning. J Sex Marital Ther.

[54] Pascoal PM, Sanchez T, Fonseca Raposo C, Pechorro P (2016). Initial validation of the Sexual Pleasure Scale in clinical and non-clinical samples of partnered heterosexual people. J Sex Med.

[55] Gravel EE, Pelletier LG, Reissing ED (2016). “Doing it” for the right reasons: validation of a measurement of intrinsic motivation, extrinsic motivation, and amotivation for sexual relationships. Pers Individ Dif.

[56] Kotiuga J, Yampolsky MA, Martin GM (2022). Adolescents’ perception of their sexual self, relational capacities, attitudes towards sexual pleasure and sexual practices: a descriptive analysis. J Youth Adolesc.

[57] Turchik JA, Garske JP (2009). Measurement of sexual risk taking among college students. Arch Sex Behav.

[58] Benassi M, Garofalo S, Ambrosini F, Sant’Angelo RP, Raggini R, De Paoli G (2020). Using two-step cluster analysis and latent class cluster analysis to classify the cognitive heterogeneity of cross-diagnostic psychiatric inpatients. Front Psychol.

[59] Milligan GW, Cooper MC (1985). An examination of procedures for determining the number of clusters in a data set. Psychometrika.

[60] Iannaccone M, D’Olimpio F, Cella S, Cotrufo P (2016). Self-esteem, body shame and eating disorder risk in obese and normal weight adolescents: a mediation model. Eat Behav.

[61] O’Loghlen E, Grant S, Galligan R (2022). Shame and binge eating pathology: a systematic review. Clin Psychol Psychother.

[62] Harrison C, Mond J, Rieger E, Rodgers B (2015). Generic and eating disorder-specific impairment in binge eating disorder with and without overvaluation of weight or shape. Behav Res Ther.

[63] Benassi VA, Sweeney PD, Dufour CL (1988). Is there a relation between locus of control orientation and depression?. J Abnorm Psychol.

[64] Duarte C, Pinto-Gouveia J, Ferreira C (2017). Ashamed and fused with body image and eating: Binge eating as an avoidance strategy. Clin Psychol Psychother.

[65] Dunkley CR, Brotto LA (2021). Disordered eating and body dissatisfaction associated with sexual concerns in undergraduate women. J Sex Marital Ther.

[66] Castellini G, D’Anna G, Rossi E, Cassioli E, Appignanesi C, Monteleone AM, Rellini AH, Ricca V (2020). Dysregulated sexuality in women with eating disorders: the role of childhood traumatic experiences. J Sex Marital Ther.

[67] Smolak L, Murnen SK (2002). A meta-analytic examination of the relationship between child sexual abuse and eating disorders. Int J Eat Disord.

[68] Cavazos-Rehg PA, Krauss MJ, Spitznagel EL, Schootman M, Bucholz KK, Peipert JF, Sanders-Thompson V, Cottler LB, Bierut LJ (2009). Age of sexual debut among US adolescents. Contraception.

[69] Holcomb BM, Mahoney CT, Lawyer SR, O’Donohue WT, Schewe PA (2019). Impulsivity and sexual assault. Handbook of sexual assault and sexual assault prevention.

[70] Wenninger K, Heiman JR (2005). Relating body image to psychological and sexual functioning in child sexual abuse survivors. J Trauma Stress.

[71] Slavin MN, Scoglio AAJ, Blycker GR, Potenza MN, Kraus SW (2020). Child Sexual abuse and compulsive sexual behavior: a systematic literature review. Curr Addict Rep.

[72] Wang ZY, Hu M, Yu TL, Yang J (2019). The relationship between childhood maltreatment and risky sexual behaviors: a meta-analysis. Int J Environ Res Public Health.

[73] Abajobir AA, Kisely S, Maravilla JC, Williams G, Najman JM (2017). Gender differences in the association between childhood sexual abuse and risky sexual behaviours: a systematic review and meta-analysis. Child Abuse Negl.

[74] Sarwer DB, Durlak JA (1996). Chidlhood sexual absue as a predictor of adult female sexual dysfunction: a study of couples seeking sex therapy. Child Abuse Negl.

[75] Gonidakis F, Kravvariti V, Varsou E (2015). Sexual function of women suffering from anorexia nervosa and bulimia nervosa. J Sex Marital Ther.

[76] Schembri C, Evans L (2008). Adverse relationship processes: the attempts of women with bulimia nervosa symptoms to fit the perceived ideal of intimate partners. Eur Eat Disord Rev.

[77] Dworkin E, Javdani S, Verona E, Campbell R (2014). Child sexual abuse and disordered eating: the mediating role of impulsive and compulsive tendencies. Psychol Violence.

[78] Castellini G, Lo Sauro C, Ricca V, Rellini AH (2017). Body esteem as a common factor of a tendency toward binge eating and sexual dissatisfaction among women: the role of dissociation and stress response during sex. J Sex Med.

[79] American Psychiatric Association (2013). Diagnostic and statistical manual of mental disorders.

[80] Bleck JR, DeBate RD, Olivardia R (2015). The comorbidity of ADHD and eating disorders in a nationally representative sample. J Behav Health Serv Res.

[81] Brewerton TD (2019). An overview of trauma-informed care and practice for eating disorders. J Aggression Maltreatment Trauma.

